# Efficacy of Acupuncture in Alleviating Negative Emotions in Polycystic Ovary Syndrome: A Systematic Review and Meta‐Analysis

**DOI:** 10.1002/brb3.71129

**Published:** 2025-12-17

**Authors:** Lanfeng Lai, Zhennan Wu, Jiayi Zhao, Jiahuan Li, Boxiong Wu, Han Yang, Nenggui Xu, Wei Yi

**Affiliations:** ^1^ South China Research Center for Acupuncture and Moxibustion, Medical College of Acu‐Moxi and Rehabilitation Guangzhou University of Chinese Medicine Guangzhou China; ^2^ Second Clinical Medical College Guangzhou University of Chinese Medicine Guangzhou China

**Keywords:** acupuncture, meta‐analysis, negative emotions, polycystic ovary syndrome

## Abstract

**Introduction:**

Preliminary studies have indicated that acupuncture may play a role in the management of negative emotions in patients with PCOS. However, the available evidence is predominantly of low quality. Consequently, a meta‐analysis was conducted to assess the validity of acupuncture as a therapeutic modality for treating negative emotional symptoms in patients with PCOS.

**Methods:**

A meticulous search of nine databases was performed to identify randomized controlled trials assessing the effectiveness of acupuncture in the management of negative emotions in individuals diagnosed with PCOS. The principal outcome was evaluated using the SDS and SAS scales, while secondary outcomes encompassed the Rosenfield scores, Ferriman–Gallwey (F–G) scores, and BMI. The risk of bias was appraised with the Cochrane RoB 2.0 tool. The certainty of evidence was evaluated using the GRADE (Grading of Recommendations Assessment, Development and Evaluation) system.

**Results:**

This meta‐analysis synthesized the findings of seven studies that collectively provided evidence supporting the hypothesis that acupuncture may be more effective than drug treatment in alleviating SDS. However, the findings did not demonstrate significant efficacy in improving SAS. The analysis further revealed that acupuncture could effectively improve the F–G score in patients with PCOS, although it was not significantly effective for the Rosenfield score. The GRADE assessment assigned a low level of certainty to the SDS and F–G scores and a very low level of certainty to the SAS, Rosenfield, and BMI scores, emphasizing the necessity for additional high‐quality RCTs to validate these findings.

**Conclusion:**

This study identified the potential of acupuncture in alleviating depression in patients with PCOS. Nonetheless, the evidence substantiating these findings was restricted by the poor quality of the included RCTs, precluding the drawing of definitive conclusions. Until more substantial evidence emerges, caution should be exercised regarding assertions of the therapeutic potential of acupuncture for treating negative emotions associated with PCOS.

## Graphical abstract

This meta‐analysis of seven RCTs (*n* = 489) demonstrates that acupuncture significantly improves depression scores, hirsutism, and BMI in PCOS patients, though anxiety symptoms show no significant improvement. Evidence quality is low, warranting cautious interpretation.

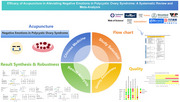

**Abbreviations**
5‐HIAA: 5‐hydroxyindoleactic acid
BL: bladder meridians
BMI: body mass index
CGRP: calcitonin gene‐related peptide
CI: confidence interval
CV: conception vessel
DA: dopamine
DHEA: dehydroepiandrosterone
DOPAC: 3,4‐dihydroxyphenylacetic acid
DRN: dorsal raphe nucleus
EA: eletroacupuncture
FSH: follicle‐stimulating hormone
GnRH: gonadotropin‐releasing hormone
GRADE: Grading of Recommendations Assessment, Development and Evaluation
HAM‐A: Hamilton Anxiety Scale
HAM‐D: Hamilton Depression Scale
HPA: hypothalamic–pituitary–adrenal
IFG.L: left inferior frontal gyrus
IMD: intermedin
LH: luteinizing hormone
MA: manual acupuncture
MD: mean difference
MEFG.L: left middle frontal gyrus
NE: norepinephrine
OCP: oral contraceptive
PCG.L: posterior cingulate gyrus
PCOS: polycystic ovary syndrome
PRISMA: Preferred Reporting Items for Systematic Reviews and Meta‐Analyses
PROSPERO: International Prospective Register of Systematic Reviews
RCT: randomized controlled trial
rs‐fMRI: resting‐state functional magnetic resonance imaging
SAS: self‐rating anxiety scale
SDS: Self‐Rating Depression Scale
TCM: traditional Chinese medicine

## 1 Introduction

Polycystic ovary syndrome (PCOS) is a complicated reproductive and metabolic disorder that affects approximately 5%–18% of females on a global scale (Joham et al. 2022). Most patients diagnosed with PCOS exhibit at least one physical symptom, including hyperandrogenemia, hirsutism, acne, irregular menstruation, infertility, insulin resistance, obesity, and dyslipidemia (Stener‐Victorin et al. 2024). PCOS frequently poses a range of clinical symptoms (Osibogun et al. 2020; Ozkan et al. 2020), with negative emotions emerging as a particularly significant complication (Cipkala‐Gaffin et al. 2012; Cooney et al. 2017; Pastore et al. 2011; Pokora et al. 2022). The prevalence of mood disorders is elevated among females diagnosed with PCOS compared to those who do not meet the criteria for the condition (Alur‐Gupta et al. 2019, 2024; Mojahed et al. 2023). The etiology of negative mood experienced by individuals diagnosed with PCOS is multifaceted and may be associated with stress, obesity, insulin resistance, elevated androgen levels, and increased cortisol levels. These effects result from disturbances in the hypothalamic (pituitary) axis (Cooney and Dokras 2017). Negative emotions have been demonstrated to exert considerable influence on the course of PCOS, exacerbating the condition and impinging on the efficacy of treatment (Zhu and Goodarzi 2022). Several studies have indicated a correlation between negative emotional states, including anxiety and depression and alterations in dietary patterns, as well as a decline in physical activity (Corfield et al. 2016; Dakanalis et al. 2023), which can contribute to further impairment of insulin sensitivity and challenges in maintaining optimal weight (Papakonstantinou et al. 2022). Therefore, mental health support should be incorporated into a comprehensive treatment plan for patients with PCOS.

The results of clinical and preclinical studies suggest a correlation between androgen excess and an elevated potential for mood disorders in offspring with PCOS (Cesta et al. 2020; Hu et al. 2015; Risal et al. 2021). Acute prenatal androgen exposure in rats has been shown to induce a PCOS phenotype, and anxiety‐like behaviors have been observed in their offspring. This may have resulted from alterations in the androgen receptor expression in the amygdala (Hu et al. 2015). Mice with PCOS, induced by dehydroepiandrosterone (DHEA), may exhibit depression‐like behavior because of alterations in monoamine and its metabolite levels within the brain (Yu et al. 2016). The dysregulation of the HPA axis associated with PCOS has been demonstrated to have the potential to contribute to the manifestation of depressive symptoms (Peecher et al. 2019; Ressler et al. 2015). Furthermore, the presence of obesity serves to exacerbate this negative mood (Karsten et al. 2021; H. Zhang et al. 2024). The contemporary therapeutic modalities for PCOS comprise a range of interventions, including lifestyle modifications, pharmacological agents, and surgical procedures (Hoeger et al. 2021). The pharmacological treatment plan includes oral contraceptives, insulin sensitizers, and psychotropic medications (Cooney and Dokras 2017). A study evaluating the efficacy of oral contraceptive treatment reported that the oral administration of 30 µg ethinyl estrogen/150 mg levonorgestrel for 6 months had minimal impact on depression and anxiety scores in patients diagnosed with PCOS (Cinar et al. 2012). In a study conducted to examine the impact of lifestyle modifications in conjunction with metformin (or placebo) in relation to oral contraception on the quality of life of patients diagnosed with PCOS, it was revealed that, while all participants exhibited an improvement in quality of life, no statistically significant differences were observed between the treatment and control groups (Harris‐Glocker et al. 2010). The 2023 International Clinical Guidelines for the Management of PCOS state that only one RCT has specifically investigated the use of antidepressants in patients diagnosed with PCOS (Masoudi et al. 2021). The guidelines recommend that antidepressants should be considered if there is little improvement with psychotherapy (Teede et al. 2023). However, a potential side effect of antidepressants is weight gain, which has the potential to exacerbate the symptoms of PCOS (Fava 2000; H. Zhang et al. 2024).

Acupuncture has been used extensively and safely worldwide for the treatment of affective disorders (Acar et al. 2013; Amorim et al. 2022; Fan et al. 2022; Sabbagh Gol et al. 2021; Yang et al. 2022) and has been used as a complementary and alternative therapeutic modality for patients with PCOS (Lim et al. 2019). Electroacupuncture enhances ovarian morphology and the motility cycle in PCOS rats by downregulating androgen levels, which modulates the kisspeptin–GnRH/LH circuit (G. Xu et al. 2023). Electroacupuncture may upregulate intermedin (IMD) in the calcitonin gene–related peptide to inhibit endoplasmic reticulum stress, thereby attenuating apoptosis and autophagy in the ovarian granulosa cells of PCOS rats (Cong et al. 2022). In addition, electroacupuncture has been demonstrated to alleviate the symptoms of PCOS by stimulating angiogenesis and increasing ovarian blood volume, thereby regulating ovarian innervation (Tong et al. 2020) and promoting follicular maturation, ovulatory processes, and corpus luteum formation (T. Ma et al. 2018). Additionally, reports indicate that electroacupuncture may enhance PCOS treatment by augmenting insulin sensitivity and regulating gene expression in the adipose tissue (Johansson et al. 2013; Kang et al. 2017; Peng, Guo, et al. 2020; Peng et al. 2020). Electroacupuncture benefits the negative mood associated with the PCOS model by downregulating hypothalamic DNA methylation (P. Cui et al. 2018) and upregulating serum estradiol and ovarian stimulating hormone receptor alpha expression (Xu et al. 2022). Several studies have shown that acupuncture therapy can facilitate ovulation, reduce androgen levels in the body, and alleviate negative emotions in patients diagnosed with PCOS (X. Chen et al. 2022; Jo and Lee 2017; Liu et al. 2023; H. Yang et al. 2023; L. Yang et al. 2023). However, there has been no comprehensive, systematic methodological evaluation or data integration of acupuncture for treating negative emotions in patients with polycystic ovaries. The purpose of this study was to conduct a systematic review and meta‐analysis of acupuncture as a treatment for negative emotions in patients with PCOS.

## 2 Methods

### 2.1 Study Registration

This study was conducted in accordance with the Preferred Reporting Items for Systematic Reviews and Meta‐Analyses (PRISMA) guidelines, adhering to the PRISMA checklist (Page et al. 2021). The study was officially endorsed by the International Prospective Register of Systematic Reviews on May 28, 2024 (registration number: CRD42024549055).

### 2.2 Search Strategy

A comprehensive literature search was conducted before May 25, 2024, using nine databases. English‐language databases included the Cochrane Library, Science Direct Search, PubMed, Embase, and Web of Science, whereas Chinese‐language databases included China Biology Medicine, VIP, Wanfang Data, and CNKI. The search was conducted comprehensively, encompassing literature from all countries and languages and comprising various article types. The keywords used in the search included “polycystic ovary syndrome,” “anxiety,” “depression,” “acupuncture,” and “randomized controlled trials.” The comprehensive search methodology is described in the supporting information.

### 2.3 Criteria for Inclusion and Exclusion

Two researchers (LF Lai and JY Zhao) independently screened the retrieved articles.

The titles and abstracts of retrieved articles were reviewed. Duplicate or irrelevant studies were excluded from the analysis. In accordance with the established criteria, studies deemed suitable for inclusion were identified, and the data were extracted and cross‐checked. Any ambiguities were resolved through discussions and consensus. In the event of disagreement, a third researcher (ZN Wu) was asked to adjudicate. The exclusion criteria were duly documented. The studies included in this review compared acupuncture (alone or in combination with other treatments) with pharmacological treatments, non‐pharmacological treatments, or an ineffective control group (including placebo, waiting list, and blank control).

The specific inclusion criteria were as follows:

- Participants must have been diagnosed with PCOS.
- The only intervention used was acupuncture. Moxibustion treatments include a range of techniques, such as manual acupuncture, electroacupuncture, and auricular acupuncture.
- The comparison group consisted of individuals who received medication or other non‐pharmacological treatments or were deemed to be in the ineffective treatment group, which included those who received a placebo, were placed on a waiting list, or did not receive any treatment.
- Outcome: The principal outcome measure was mental health, as determined by anxiety scales (HAM‐A and SAS), depression scales (HAM‐D and SDS), and cognitive impairment scales.
- Study design: This was an RCT.

The following criteria resulted in exclusion from the study:

- 1. Non‐PCOS
- 2. Nonrandomized controlled clinical study design
- 3. Comparison between different types of acupuncture
- 4. A literature review
- 5. The study did not describe results on depression, anxiety, or cognitive impairment.
- 6. Dataset replication
- 7. Acupuncture in conjunction with pharmacological agents.

### 2.4 Data Collection Process

A preliminary screening of the titles, abstracts, and keywords was conducted by two researchers (JH Li and BX Wu) to exclude duplicates and documents that did not conform to the pre‐established criteria for inclusion. Subsequently, a thorough examination was performed to ascertain the compliance of the remaining documents with the stipulated inclusion criteria. The documents were then collated and assigned identification numbers using EndNote X9 software, and the data were organized according to the following parameters: author, year of publication, patient age, patient type, intervention methodology, acupuncture point and electroacupuncture configuration, duration of continuous treatment, source of PCOS diagnosis, primary outcome, and adverse events.

### 2.5 Assessment of Bias Risk

The Cochrane Rob 2.0 was utilized to evaluate the validity of the extant literature and the risk of bias inherent in the included studies (Sterne et al. 2019). Each RCT was evaluated according to the following six criteria: random sequence generation, unintended consequences, missing primary outcome data, measurement of the primary outcome, selective reporting, and overall assessment. The classification of studies was determined as follows: low risk when the research methods were appropriate, clearly and precisely described, and high risk when the methods were unclear or problematic. Two researchers (LF Lai and JY Zhao) independently assessed the studies, and in the event of disagreement, a third researcher (ZN Wu) resolved the issue.

### 2.6 Statistical Analysis

The included RCTs were submitted to statistical analysis using the R “metafor” software package. Continuous data were expressed as mean difference (MD) or standardized mean difference with 95% confidence intervals (CI). To evaluate heterogeneity, *I* ^2^ and *p* values were considered. Sensitivity analyses were deemed necessary when the *I* ^2^ value exceeded 50%, or the *p <* 0.05, indicating substantial heterogeneity. Furthermore, subgroup analyses were used to identify sources of heterogeneity, with a random‐effects model utilized when necessary and a fixed‐effects model when *p* > 0.05 and *I* ^2^ < 50%.

## 3 Results

### 3.1 Study Characteristics

**FIGURE 1:**
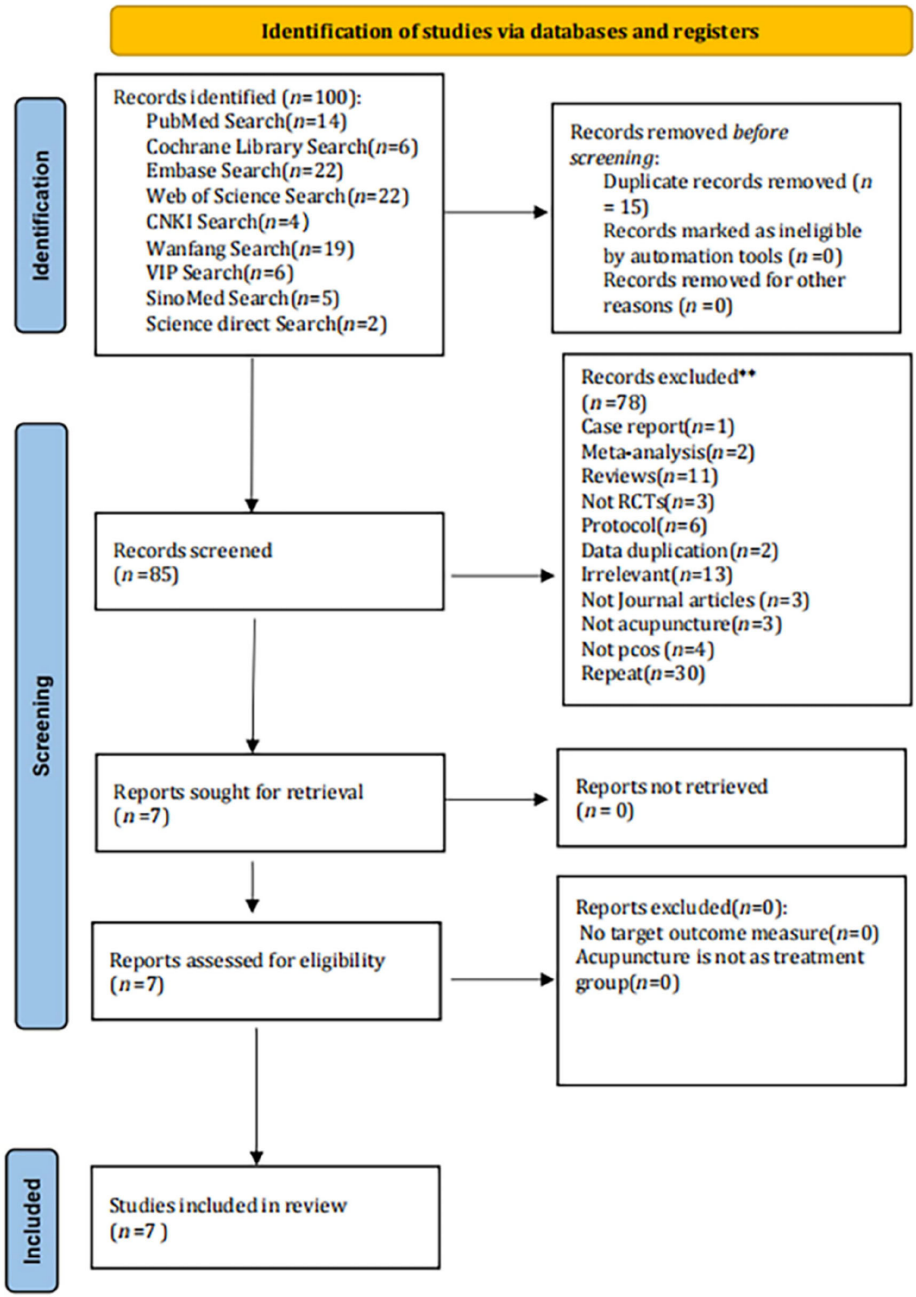
Preferred Reporting Items for Systematic Reviews and Meta‐Analyses (PRISMA) flowchart of the included studies.

In accordance with the specified search strategy, a corpus of 100 articles was obtained, comprising six from the Cochrane Library, 14 from PubMed, 22 from Web of Science, 2 from ScienceDirect, 5 from SinoMed, 22 from Embase, 4 from CNKI, 6 from Wipro, and 19 from Wanfang. Following a thorough review of the titles and abstracts, 15 duplicate articles were excluded, and 78 additional articles were excluded after a full‐text review. The seven articles included in this review are shown in Figure 1 (Jin et al. 2016; H. Ma et al. 2016; Mao and Lin 2021; D. Wu et al. 2023; Yao et al. 2018; Zhang et al. 2020; S. K. Zhang et al. 2024).

Table 1 provides an overview of the fundamental characteristics of these studies. The mean ages at baseline, which were considered pre‐intervention data, were examined. The post‐intervention dataset comprised intervention, treatment processes, and adverse events. The only explicitly reported age range was 20‐40 years (H. Ma et al. 2016), while the remaining five studies presented data as mean ± standard deviation, with means ranging from approximately 21 to 30 years. One study did not report the age of its participants (Mao and Lin 2021). The seven studies were all RCTs conducted in China, with 489 patients suffering from PCOS included in the research. Detailed characteristics of each study are presented in Table 1

**TABLE 1:** Characteristics of the included studies.

References	Age	Intervention	Sample size (T:C)	IG	CG	Duration (weeks)	Frequency	Outcome	Acupoints	Diagnose
H. Ma (2016)	20–40	MA	57 (29:28)	MA + clomiphene	Clomiphene	12	MA: t.i.w.; control: q.d. on days 1–5 of menstrual cycle, repeat for three cycles.	SAS	BL18, BL23, CV12, CV9, CV4, CV3	①
C. L. Jin (2016)	29 ± 4/28 ± 4	EA	68 (35:33)	EA	Diane‐35	12	EA: t.i.w.; control: q.d. from the fifth day of menstrual cycle for 21days, repeat for three cycles.	SCL‐90, TCM symptom score	BL18, CV17, LR14, CV12, ST25, CV4, EX‐CA1, SP6, ST36, LR3	①
S. K. Zhang (2024)	28.99 ± 3.58/29.59 ± 3.76	EA	60 (30:30)	Transcranial vagal electrical stimulation + lifestyle intervention	Lifestyle intervention	12	EA: t.i.w.; control: q.d.	SAS, SDS, BMI, TCM symptom score	Select 2 points in each bilateral tragus	①②
M. Y. Mao (2021)	/	EA	108 (54:54)	EA + Metformin hydrochloride, Diane‐35	Metformin hydrochloride, Diane‐35	12	EA: q.o.d.; control: Metformin hydrochloride t.i.d., Diane‐35 q.d. from the third day of menstrual cycle for 21 days, repeat for three cycles.	BMI, SAS, SDS	GV20, GV29, BL20, BL21, BL15, ST25, CV4, CV6, ST29,	①
									CV12, LI4, LR3, ST36,	
									SP6	
H. L. Zhang (2020)	29 ± 2/28 ± 3	EA	40 (20:20)	EA + lifestyle intervention	Lifestyle intervention	16	EA: t.i.w.; control: q.d.	BMI, F‐G score, SDS, SAS, PCOSQ,	GV20, CV12, CV4, ST29, ST32, ST34, SP6, ST36, HT7, LI4	①
D. Wu (2023)	21.89 ± 9.71/22.33 ± 9.49	MA	60 (30:30)	MA	Letrozole	12	MA: q.d. for 14 consecutive days each month, for a total of 3 months; control: q.d. from Day 5 of the menstrual cycle for 5 days; repeat for 3 months	SAS, SDS	GV20, CV4, BL23, BL18, BL20, EX‐CA1, SP6	③
M. Yao (2018)	27. 8 ± 4.8/28. 2 ± 4.5	EA	96 (48:84)	EA	Metformin	24	EA: t.i.w.; control: t.i.d.	BMI, Rosenfield score, F‐G score, SAS	CV17, BL18, ST25, EX‐CA1, ST36, LR14, CV12, CV4, SP6, LR3	①

Abbreviations: CG, Controlled Group; EA, electroacupuncture; IG, Interventional Group; MA, manual acupuncture; q.d., once a day; q.o.d., every other day; t.i.d., three times a day; t.i.w., three times a week.

①: National diagnostic criteria were generated in Rotterdam in May 2003; ②: 2018 Chinese Medical Association's Obstetrics and Gynecology Polycystic Ovary Syndrome Diagnosis and Treatment Guidelines; ③: 2012 PCOS diagnostic criteria of the Chinese Medical Association's Obstetrics and Gynecology, Endocrinology branch.

The control group in seven trials received routine treatment according to the study protocol. Two studies used lifestyle interventions (Zhang et al. 2020; S. K. Zhang et al. 2024), whereas the control groups in the remaining five trials utilized pharmacological treatments, namely Diane‐35, metformin hydrochloride, letrozole, and clomiphene (Jin et al. 2016; H. Ma et al. 2016; Mao and Lin 2021; D. Wu et al. 2023; Yao et al. 2018). In one trial, the treatment group received acupuncture alone (D. Wu et al. 2023). In two trials, the treatment group received electroacupuncture (Jin et al. 2016; Yao et al. 2018). In two additional trials, the treatment group received electroacupuncture combined with medications (H. Ma et al. 2016; Mao et al. 2021). Two trials used acupuncture (electroacupuncture and auricular acupuncture) combined with lifestyle interventions (Zhang et al. 2020; S. K. Zhang et al. 2024).

Three trials documented the occurrence of adverse events (Jin et al. 2016; Yao et al. 2018; S. K. Zhang et al. 2024). The most commonly reported adverse events in patients undergoing acupuncture are localized discomfort after needle removal and minor subcutaneous bleeding. One trial (S. K. Zhang et al. 2024) reported three patients with minor subcutaneous bleeding after needle removal in the treatment group, which was relieved by local pressure. One trial (Jin et al. 2016) reported two patients with adverse reactions in the control group. One trial documented a single instance of mild subcutaneous bleeding following acupuncture in the treatment group. In contrast, the control group exhibited seven patients with gastrointestinal reactions, one patient each with headache and rash, and two patients with poor appetite (Yao et al. 2018).

### 3.2 Risk of Bias Assessment

**FIGURE 2:**
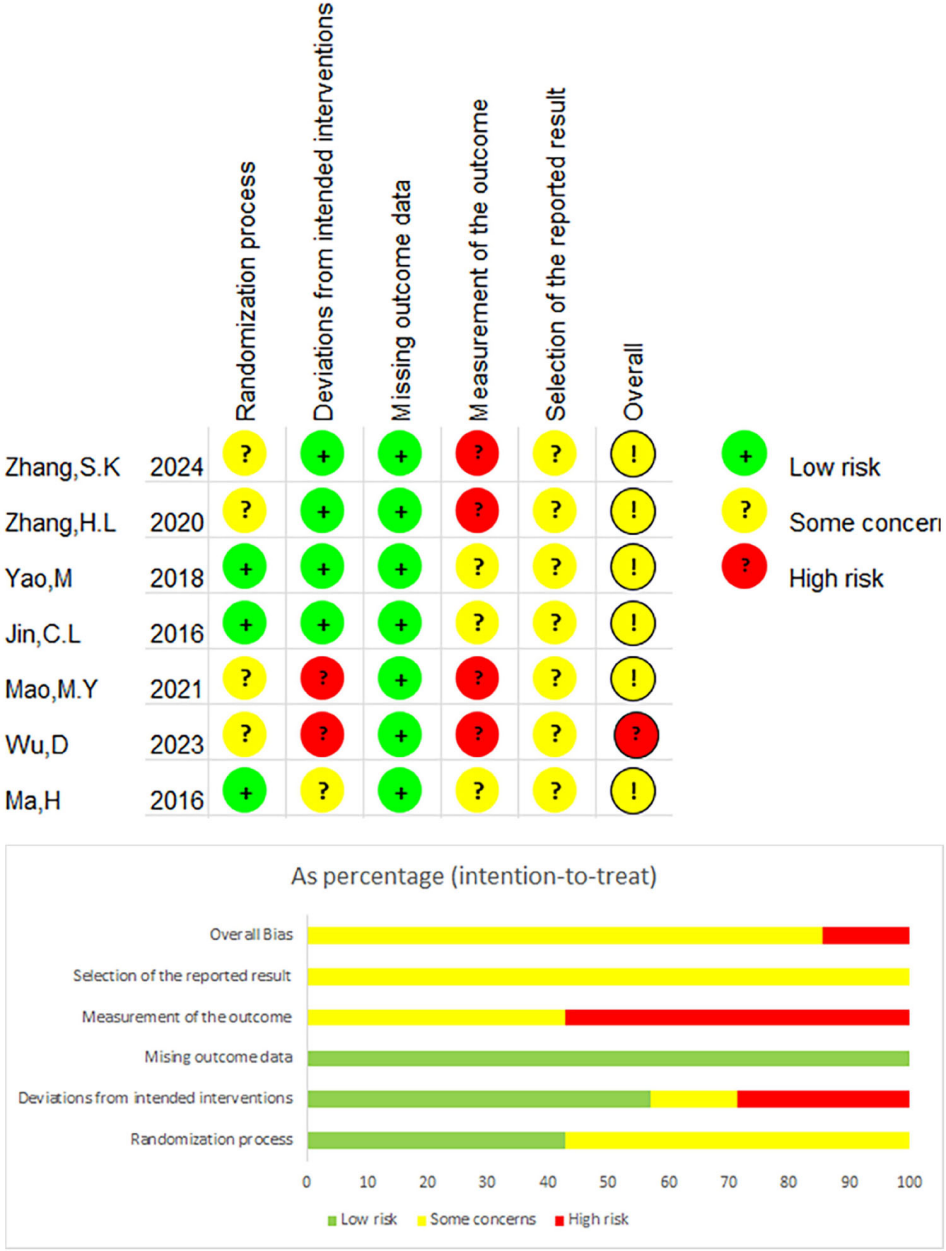
Assessment of the risk of seven studies.

The Cochrane Rob2.0 tool was used to evaluate the inherent risk of bias in individual studies (Sterne et al. 2019). The following six categories were extracted from each randomized controlled trial (Zhang et al. 2020; S. K. Zhang et al. 2024; Yao et al. 2018; Jin et al. 2016; Mao and Lin 2021; D. Wu et al. 2023; H. Ma et al. 2016) for assessment: (1) randomization process, (2) deviations from the intended interventions, (3) missing outcome data, (4) measurement of outcomes, (5) selective reporting of results, and (6) overall assessment. The study was deemed to be at minimal risk when an appropriate methodology was used, and relevant procedures were documented objectively (Figure 2).

### 3.3 Results of the Meta‐Analysis

#### 3.3.1 SAS

**FIGURE 3:**
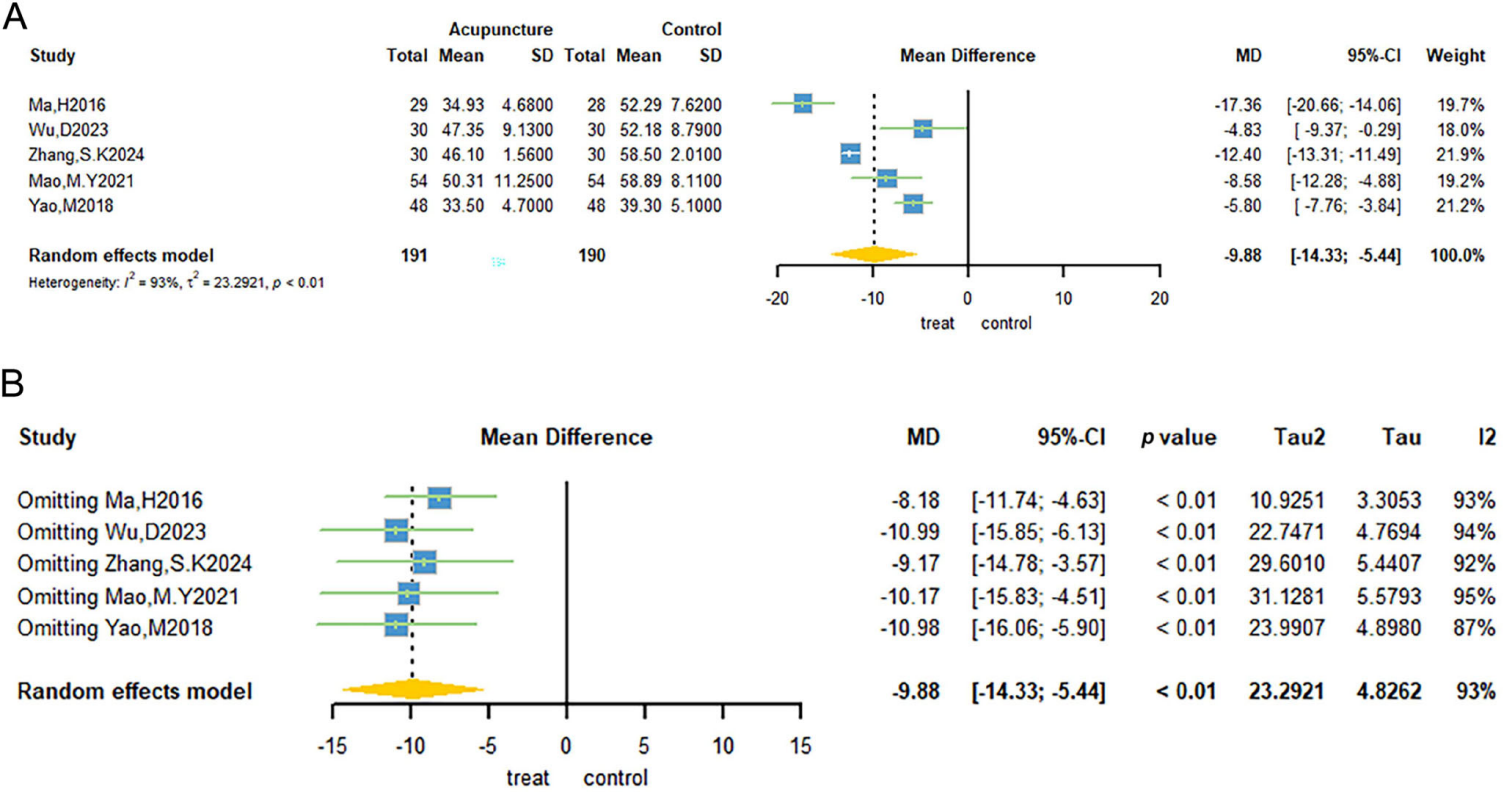
Five studies evaluated SAS of acupuncture treatment for PCOS forest plots.

Five trials (H. Ma et al. 2016; Mao and Lin 2021; D. Wu et al. 2023; Yao et al. 2018; S. K. Zhang et al. 2024) reported the use of the SAS as an outcome measure. A cohort of 381 patients was enrolled in the study, with 191 patients assigned to the acupuncture group and 190 to the non‐acupuncture control group. The meta‐analysis data indicated that the weighted mean difference in SAS was −9.88 (95% CI: −14.33; −5.44; Figure 3A). The findings demonstrated a considerable degree of heterogeneity (Tau^2^ = 23.2921, *p* < 0.01, and *I* ^2^ = 93%; Figure 3B). A subsequent sensitivity analysis was conducted; however, heterogeneity persisted, suggesting that acupuncture exerted a negligible influence on the enhancement of SAS scores in patients diagnosed with PCOS.

#### 3.3.2 SDS

**FIGURE 4:**
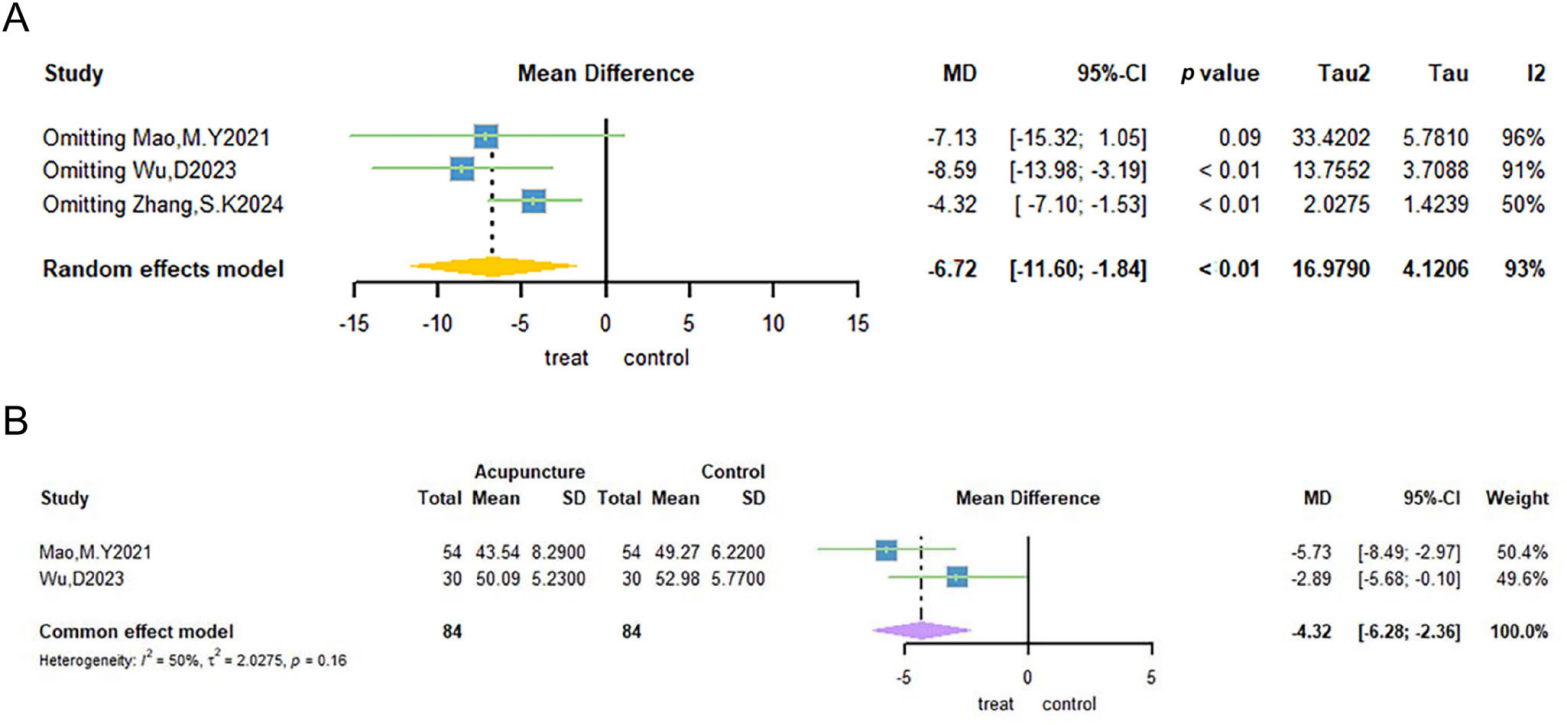
Forest plot of SDS for acupuncture treatment of PCOS.

Three trials (Mao and Lin 2021; D. Wu et al. 2023; S. K. Zhang et al. 2024) used SDS as an outcome measure. A total of 168 patients were included in the study: 84 each in the acupuncture group and 84 in the non‐acupuncture group. The results of the meta‐analysis indicated that the weighted mean difference in the SDS was −6.72 (95% CI: −11.60; −1.84; Figure 4A). The findings demonstrated a considerable degree of heterogeneity (Tau^2^ = 16.9790, *p* < 0.01, and *I* ^2^ = 93%; Figure 4A). To determine the source of heterogeneity, a sensitivity analysis was performed. Heterogeneity decreased significantly as the analysis progressed, with the *I* ^2^ value dropping to 50% after removing one study. The analysis was then recombined using a fixed‐effects model, which demonstrated that acupuncture treatment could reduce depression scores in patients with PCOS (MD: −4.32, 95% CI: −6.28 to −2.36; Figure 4B).

#### 3.3.3 Ferriman–Gallwey score

**FIGURE 5:**
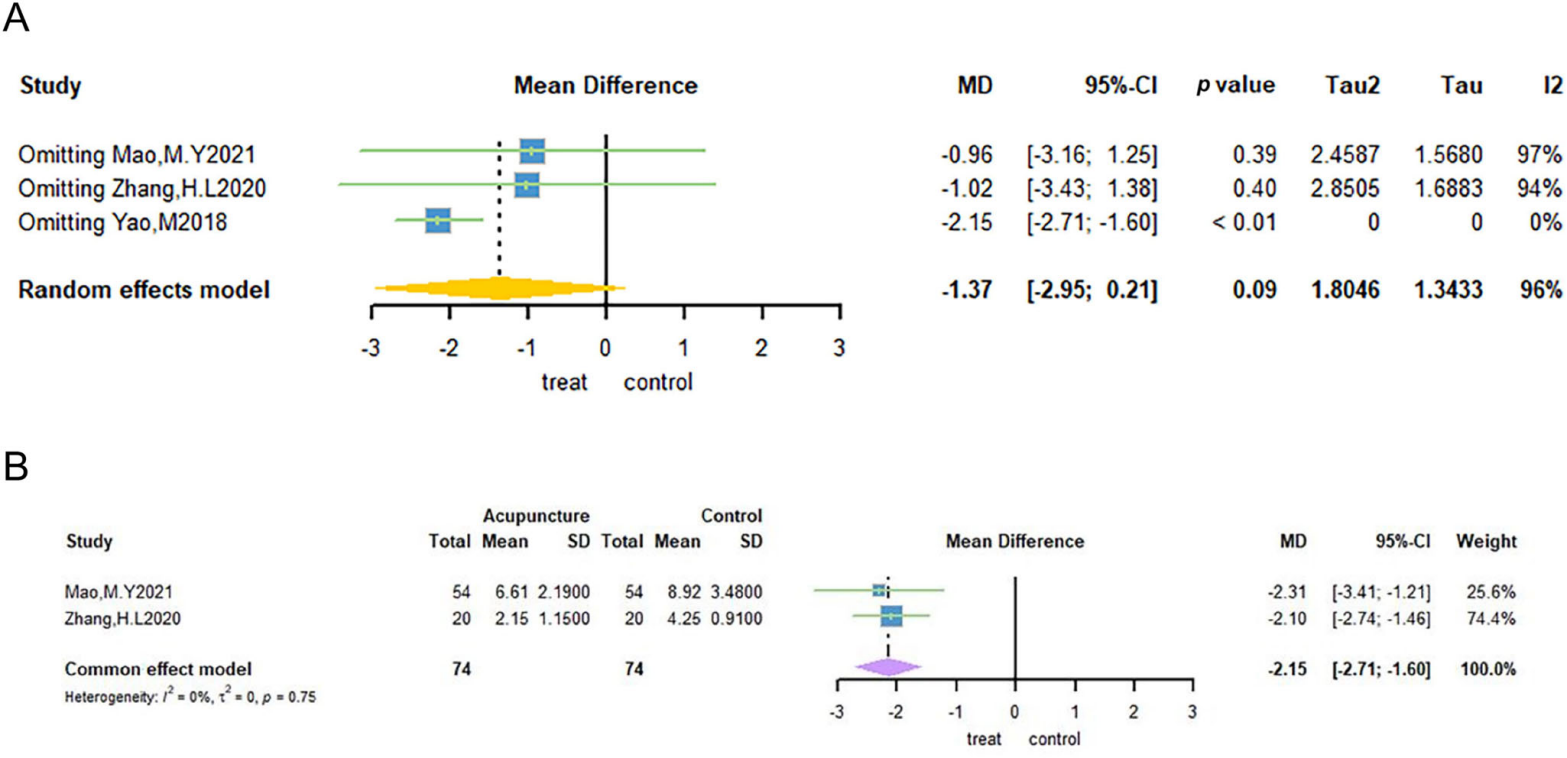
Forest plot of Ferriman–Gallwey scores for acupuncture treatment of PCOS.

The Ferriman–Gallwey (F‐G) score was used as an outcome measure in three trials (Mao and Lin 2021; Yao et al. 2018; Zhang et al. 2020). A sample of 148 patients was included in the study, with 74 patients assigned to the acupuncture group and 74 to the non‐acupuncture group. The meta‐analysis demonstrated that the weighted mean difference in F–G scores was −1.37 (95% CI: −2.95; 0.21; Figure 5A). The analysis revealed a substantial degree of heterogeneity (*I* ^2^ = 96%; Tau^2^ = 1.8046, *p* = 0.09; Figure 5A). To further clarify the source of the high heterogeneity, one trial was excluded through sensitivity analysis (Yao et al. 2018). This resulted in no heterogeneity between the two groups (*I* ^2^ = 0%; Figure 5B). The current used a fixed‐effect model, which indicated that acupuncture is an efficient method for alleviating hirsutism in patients diagnosed with PCOS (MD: −2.15, 95% CI: −2.71 to −1.6; Figure 5B).

#### 3.3.4 BMI

**FIGURE 6:**
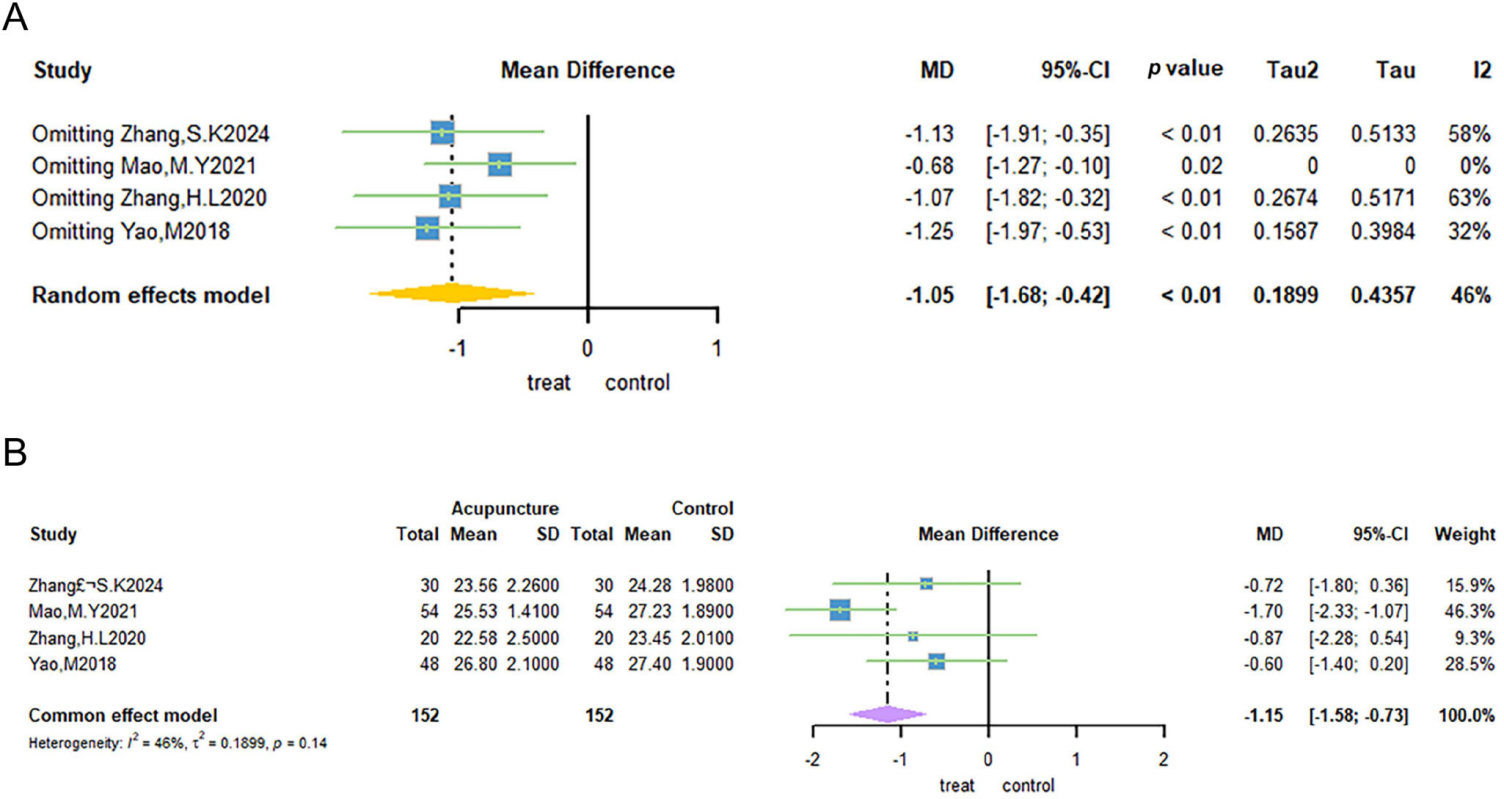
Forest plot of BMI for acupuncture treatment of PCOS.

Four trials (Mao and Lin 2021; Yao et al. 2018; Zhang et al. 2020; S. K. Zhang et al. 2024) used BMI as the outcome indicator. A total of 304 patients were selected for inclusion in the study, with 152 receiving acupuncture treatment and 152 not receiving it. The meta‐analysis demonstrated that the weighted mean difference in BMI was −1.15 (95% CI: −1.58; −0.73; Figure 6B). The two groups exhibited minimal heterogeneity (*I* ^2^ = 46%; Figure 6A) when a fixed‐effects model was used, suggesting that acupuncture treatment can reduce the BMI of patients with PCOS (MD: −1.15, 95% CI: −1.58 to −0.73; Figure 6B).

#### 3.3.5 GRADE

**FIGURE 7:**
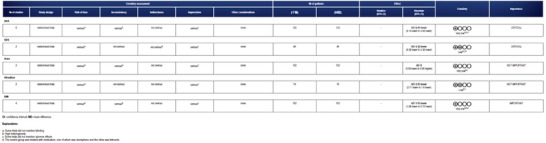
GRADE evidence.

This review used the GRADE (Grading of Recommendations Assessment, Development and Evaluation) system to evaluate the effectiveness of acupuncture in ameliorating symptoms, including depression, hirsutism, acne, and BMI. The review method involved a comprehensive analysis of seven randomized controlled trials, which collectively provided substantial evidence for the efficacy of acupuncture in reducing depression levels, with an observed mean difference of −4.2 (95% CI: −6.28; −2.36; Figure 4B). However, the GRADE downgrading rules identified issues with the treatment effect in the acupuncture group in five articles and high heterogeneity. Additionally, several intervals on the SDS score improvement scale were substantial; however, the study's substantial sample size ensured the credibility of the evaluation. Consequently, evidence supporting this finding was considered low. Consequently, the SAS, Rosenfield score, and BMI were assigned a low rating, while the F–G score was assigned a low rating, as illustrated in Figure 7.

## 4 Discussion

PCOS is a heterogeneous disease often accompanied by menstrual disorders, infertility, obesity, hirsutism, and acne. These comorbidities may increase psychological stress in patients (Teede et al. 2023). Negative emotions associated with PCOS may be related to neuroendocrine changes, including changes in sex hormones, insulin resistance, abnormal monoamine regulation, abnormalities in the HPA axis, and chronic inflammation (Cooney and Dokras 2017). Recent years have witnessed an increase in research in this domain, demonstrating that aberrant monoamine neurotransmission plays a pivotal regulatory role in DHEA‐induced depression‐like behavior in mice with PCOS. Neurotransmitter testing revealed significantly lower levels of norepinephrine (NE) and 5‐hydroxyindoleacetic acid (5‐HIAA) in the hippocampal region of model mice, and significantly lower levels of NE, 5‐HIAA, dopamine, and its metabolite, DOPAC, in the hypothalamus (Yu et al. 2016). This finding suggests that metabolic disorders of the central monoamine neurotransmitters may underlie the neurochemical basis of the depressive phenotype associated with PCOS. Manti et al. (2018) constructed a model of offspring with PCOS fed a high‐fat diet, combined with prenatal androgen exposure, and demonstrated that the offspring exhibited a significant anxiety‐like behavioral phenotype. Mechanistic studies suggest that this phenomenon is closely related to overactivation of the central noradrenergic system and dysfunction of the HPA axis. Subsequent studies demonstrated that the cortisol stress response is diminished in animal models of PCOS. Using a comprehensive metabolic and behavioral analysis, Ressler et al. (2015) substantiated that this aberrant neuroendocrine regulation may serve as a pivotal pathological mechanism underlying anxiety‐like behavior in PCOS. Li et al. (2020) used resting‐state functional magnetic resonance imaging to determine that the strength of functional connectivity between the left middle frontal gyrus and the left inferior frontal gyrus in patients with PCOS exhibited a significant and positive correlation between serum testosterone levels and the strength of functional connectivity between the left posterior cingulate gyrus and the left insula gyrus, whereas a negative correlation was observed between luteinizing hormone (LH) levels and the strength of functional connectivity between these regions. Identifying these novel neuroimaging biomarkers provides substantial insights into the neural circuit mechanisms underlying mood disorders in PCOS.

In recent years, acupuncture has made significant progress in the field of research on the neurobiological mechanisms regulating PCOS and negative emotions. Studies have demonstrated that acupuncture exerts neurobiological effects through multiple target regulatory pathways. First, at the level of neurotransmitter regulation, Han et al. (2018) confirmed that electroacupuncture could effectively reverse synaptic plasticity damage in the CA1 region of the hippocampus by regulating 5‐hydroxytryptamine (5‐HT) receptor subtypes. The underlying mechanism involves the restoration of glutamatergic synaptic long‐term potentiation and inhibition of abnormal long‐term depression. Second, regarding neuroimmune interactions, Song and Wang (2011) proposed that inflammatory factors can affect the metabolism of monoamine neurotransmitters through the blood–brain barrier, leading to an imbalance in 5‐HT and NE neurotransmission in the prefrontal‐limbic system. Acupuncture has been demonstrated to exert a substantial inhibitory effect on pro‐inflammatory factors, including IL‐6 and TNF‐α (Cai et al. 2019). Yang et al. (2021) further revealed that this anti‐inflammatory effect is intimately associated with the vagus nerve–cholinergic anti‐inflammatory pathway, which is mediated by the activation of the α7 nicotinic acetylcholine receptor.

Regarding neuroendocrine regulation (J. Cui et al. 2021), studies have demonstrated that acupuncture exerts bidirectional regulatory properties on the HPA axis. Harbach et al. (2007) observed in a clinical experiment that acupuncture intervention significantly reduced plasma cortisol concentrations (*p* < 0.05). Concurrently, electroacupuncture has been observed to activate the cannabinoid CB2 receptor, thereby inducing a substantial upregulation of POMC peptide and β‐endorphin expression (Su et al. 2011). StenerVictorin et al. (2008) observed that this neuropeptide‐mediated effect could restore the pulsatile secretion of gonadotropin‐releasing hormone (GnRH) in the hypothalamus and normalize the LH/follicle‐stimulating hormone ratio in a PCOS model.

The modulatory effects of acupuncture on emotion‐related neural circuits are an interesting area of research. Y. Chen et al. (2024) posited that electroacupuncture might exert an anxiolytic effect through the BLA^CaMKII^‐rACC neural circuit via chemogenetic techniques. As demonstrated by Z. Wu et al. (2024), the anxiolytic effect of electroacupuncture in rats with inflammatory pain is contingent on the activation of rACC CAMKII neurons projecting to the DRN serotonergic neurons. These observations provide an experimental basis for explaining the multidimensional neuroimmune–endocrine regulatory network of acupuncture in the treatment of mood disorders in PCOS. Although preliminary studies suggest that acupuncture may have anxiolytic effects by modulating neural circuits (Y. Chen et al. 2024; Z. Wu et al. 2024), this meta‐analysis did not observe a significant improvement in anxiety symptoms among patients with PCOS. This may be attributable to the acupuncture protocol employed in the study (including acupoint selection and stimulation parameters) potentially being insufficient to effectively activate the aforementioned anti‐anxiety neural circuits. Furthermore, anxiety in PCOS patients exhibits multifactorial and heterogeneous pathophysiological characteristics, whose complexity may exceed the regulatory scope of current acupuncture interventions. Consequently, future clinical trials should be informed by robust preclinical mechanistic research in order to design more precise protocols targeting the core features of PCOS‐related anxiety.

This study is the first systematic evaluation of the therapeutic effect of acupuncture on negative emotions associated with PCOS through a systematic review and meta‐analysis of seven RCTs (Jin et al. 2016; H. Ma et al. 2016; Mao and Lin 2021; D. Wu et al. 2023; Yao et al. 2018; Zhang et al. 2020; S. K. Zhang et al. 2024) (involving 509 participants). The findings demonstrated that acupuncture exhibited a substantial benefit over conventional drug therapy in enhancing SDS scores (MD = −4.32, 95% CI [−6.28, −2.36]) and concomitantly exerted a more pronounced effect in alleviating hirsutism (MD = −2.15, 95% CI [−2.71, −1.60]) and BMI regulation (MD = −1.15, 95% CI [−1.58, −0.73]). However, no significant difference in SAS scores was observed between the acupuncture and control groups. This apparent lack of therapeutic efficacy may be attributable to a number of factors. First, methodological limitations in the original studies, such as small sample sizes, resulted in insufficient statistical power to detect subtle changes in anxiety symptoms. Additionally, the extremely high heterogeneity (*I* ^2^ = 93%) in the SAS analysis constituted a major confounding factor. Ultimately, the nature of the outcome measure itself may have contributed. In comparison to the SDS scale, the SAS may demonstrate diminished sensitivity to particular emotional fluctuations induced by acupuncture in the PCOS population. Furthermore, anxiety symptoms in PCOS patients may present with greater complexity than depressive symptoms, or demonstrate increased resistance to acupuncture treatment. These findings suggest that the therapeutic effects of acupuncture may not be uniformly consistent across all psychological dimensions. Acupuncture did not demonstrate a statistically significant difference in acne scores among patients with PCOS. This discrepancy may be attributed to the inclusion of only two articles in this review (Mao and Lin 2021; Yao et al. 2018). We could not perform a meta‐analysis because of the limited number of included studies and the inability to reduce high heterogeneity through sensitivity analysis.

We observed considerable heterogeneity in our research (*I* ^2^ = 93%–96%). This discrepancy may be attributable to variations in intervention methods and control group designs across studies. The term “acupuncture” is a broad concept that encompasses a range of distinct therapies, including traditional manual acupuncture, electroacupuncture, and auricular vagus nerve stimulation. A sensitivity analysis of the SDS outcome measure revealed a significant reduction in heterogeneity (*I* ^2^ value decreasing from a peak of 93% to 50%) when excluding the sole study employing auricular stimulation (S. K. Zhang et al. 2024). This finding suggests that the mechanism of action or effect size of auricular stimulation may differ from that of conventional body acupuncture. Moreover, inconsistencies were identified in the design of the control groups across studies. The pharmacological controls included metformin, clomiphene, Diane‐35, letrozole, and lifestyle interventions. The diversity inherent in comparative arms studies has been identified as a potential factor that has obscured the assessment of acupuncture's efficacy. Besides, the absence of standardized treatment parameters has resulted in inconsistencies within acupuncture protocols. The selection of needles is constrained by multiple approaches, while fundamental parameters such as stimulation frequency, intensity, and treatment duration (ranging from 12 to 24 weeks) remain inconsistent. This has the effect of exacerbating research heterogeneity and hindering the establishment of optimized treatment protocols that are replicable in clinical practice or subsequent studies. Consequently, the extant evidence base remains inadequate for the purpose of providing clinical guidance.

Currently, there are few studies on the efficacy of acupuncture in ameliorating negative emotions in patients with PCOS, and there is much room for exploration in this area. Second, negative emotions are subjective symptoms, and the widespread acceptance of acupuncture in the Chinese population indicates that it is susceptible to a placebo effect during treatment. Furthermore, there is currently no standardized treatment plan for alleviating negative emotions in patients with PCOS. Different acupuncture points, electroacupuncture frequencies, and amplitudes affect the results. Only two trials (Yao et al. 2018; S. K. Zhang et al. 2024) reported adverse reactions after acupuncture, namely subcutaneous bleeding and mild pain at the intervention site, confirming the safety of this procedure.

The current study is the inaugural examination of the impact of acupuncture on negative emotions in females diagnosed with PCOS. Previous meta‐analyses have investigated the effects of acupuncture on sex hormones and pregnancy rates in females diagnosed with PCOS but not on negative emotions (Jo et al. 2017). This study synthesized data from seven eligible studies (Jin et al. 2016; H. Ma et al. 2016; Mao and Lin 2021; D. Wu et al. 2023; Yao et al. 2018; Zhang et al. 2020; S. K. Zhang et al. 2024) using SAS and SDS scales to assess changes in patients' emotions. The findings of this study may provide preliminary evidence for the use of acupuncture as a non‐pharmacological treatment to alleviate negative emotions in patients with PCOS. The current findings underscore the potential of acupuncture as an alternative or complementary therapy for treating negative emotions associated with PCOS.

The majority of articles utilized points on the conception vessel (CV) and bladder meridians (BL) (Jin et al. 2016; H. Ma et al. 2016; Mao et al. 2021; D. Wu et al. 2023; Yao et al. 2018), with a significant number using Zhongwan (CV12), Guanyuan (CV4), and Ganshu (BL18). The selection of CV12, CV4, and BL18 was based on their extensive application in traditional Chinese Medicine (TCM) theory and practice, particularly in the context of emotional disorders associated with PCOS (J. Wang. 2020; J. Dai et al. 2020; Dai et al. 2023; Lin et al. 2018; H. Liu et al. 2022; J. M. Wu et al. 2020; Zeng 2019). According to the principles of TCM, the pathogenesis of PCOS is associated with deficiencies in the liver and kidneys, as well as with the mutual obstruction of phlegm and stasis. The therapeutic approach of TCM involves the regulation of the liver and kidneys with the objective of unblocking blood. The emotional distress associated with this condition is believed to originate in the heart, liver, spleen, and kidneys. The therapeutic approach of TCM for mood disorders involves a multifaceted approach encompassing the calming of the mind and stabilization of the will, alleviation of liver function, regulation of qi, and balancing of the yin and yang of blood and qi. CV12 is associated with the conception channel located in the upper abdomen. It has been observed to gather and transmit the function of local water and fluid, regulate qi in the middle energizer, and enhance the hub function. The CV4 is a conception channel located in the lower abdomen. CV4 tonifies the kidney and essence, strengthens the foundation, and nourishes the yuan. BL18, located on the back, is associated with the bladder meridian and has been shown to alleviate liver function, regulate qi, and modulate mood. The therapeutic application of acupuncture has been demonstrated to alleviate symptoms associated with PCOS, with a concomitant positive effect on the emotional well‐being of patients.

## 5 Limitations

In view of the substantial methodological limitations and inherent risk of bias in the included RCTs, the conclusions of this review must be interpreted with caution. The reliability and generalizability of the findings are collectively undermined by these factors. First, the methodological rigor of the original studies was generally low. Multiple studies failed to adequately report randomization and allocation concealment procedures (Mao and Lin 2021; D. Wu et al. 2023; Zhang et al. 2020; S. K. Zhang et al. 2024). The absence of rigorous blinding designs may substantially increase the risk of selection bias, thereby compromising baseline comparability between intervention and control groups and potentially undermining the internal validity of findings.

Next, the statistical power of the included trials is questionable. The limited sample sizes of the selected studies (Zhang et al. 2020) compromise their capacity to discern genuine effects while concomitantly elevating the likelihood of Type I (false positive) and Type II (false negative) errors. Furthermore, substantial heterogeneity was observed in the initial analyses of both the SAS and SDS, with *I* ^2^ values exceeding 90%. The marked heterogeneity observed indicates that the results of a pooled analysis may not be representative of a single underlying effect. This phenomenon may be attributed to significant variations in acupuncture protocols, including diverse needling techniques (manual, electroacupuncture, and auricular acupuncture), point selection methods, stimulation parameters (frequency and intensity), and treatment duration (ranging from 12 to 24 weeks). The clinical diversity inherent in the study design may have resulted in the inclusion of studies employing heterogeneous interventions, which may have yielded misleading conclusions and severely limited the generalizability of the findings. Consequently, existing evidence is insufficient to establish a specific effective acupuncture protocol. Finally, publication bias cannot be disregarded, as all included studies originated from China. According to the GRADE system of assessment, the quality of the evidence is low. There is an urgent need for large‐scale, methodologically rigorous randomized controlled trials to provide conclusive evidence.

## 6 Conclusion

The observations derived from this analytical process indicate that acupuncture may hold the capacity to alleviate depressive symptoms in patients diagnosed with PCOS. Nevertheless, the substantiation supporting these observations was deficient because of the overall inadequate quality of the RCTs that were incorporated into this analysis. This deficiency in the quality of the included trials resulted in the inability to draw definitive conclusions. Furthermore, these findings suggest that acupuncture does not demonstrate significant efficacy in alleviating anxiety, indicating the need for further exploration in future research. Large‐sample, multicenter, double‐blind phase III RCTs should be conducted using a standardized acupuncture protocol combined with neuroendocrine marker testing and a placebo control (e.g., superficial needling at non‐acupuncture points) to improve the specificity of the trial, and the follow‐up period should be extended to more than 24 weeks to evaluate the sustained effect of the intervention. Until more conclusive evidence emerges, caution should be exercised regarding the therapeutic potential of acupuncture for treating negative emotions associated with PCOS.

## Author Contributions

Lanfeng Lai conceived the research idea and prepared the initial draft of the manuscript. Zhennan Wu designed the structure of the article, created the figures, and provided suggestions for revisions. Jiayi Zhao and Jiahuan Li conducted comprehensive database searches and screened relevant articles. Lanfeng Lai and Boxiong Wu were responsible for data extraction. Han Yang processed the data and contributed to the revision of the manuscript. Nenggui Xu and Wei Yi supervised the entire review process. All authors critically reviewed and approved the final manuscript.

## Funding

This study was supported by the National Natural Science Foundation of China (Grant no. 82374569).

## Ethics Statement

The authors have nothing to report.

## Consent

The authors have nothing to report.

## Conflicts of Interest

The authors declare no conflicts of interest.

## Data Availability

The data supporting the findings of this study are available within the article and/or its supporting information.
